# p.R394G耐药复发急性早幼粒细胞白血病1例

**DOI:** 10.3760/cma.j.issn.0253-2727.2023.12.016

**Published:** 2023-12

**Authors:** 月 史, 晶晶 姚, 艳红 姚, 志彬 刘, 峰 高, 晓宇 李, 术青 冯

**Affiliations:** 1 华北理工大学附属医院血液内科，唐山 063000 Department of Hematology, North China University of Science and Technology Affiliated Hospital, Tangshan 063000, China; 2 唐山职业技术学院，唐山 063000 Tangshan Vocation & Technical College, Tangshan 063000, China

患者，女，因“乏力10余天”于2018年9月13日收入当地医院。血常规：WBC 0.89×10^9^/L，HGB 74 g/L，PLT 83×10^9^/L。凝血系列：D-二聚体0.901 mg/L，余正常。外周血涂片：白细胞数量少，偶见幼稚细胞，占5％，红细胞大小不等，成堆散在血小板少见。骨髓细胞形态学：增生极度活跃，异常早幼粒细胞占30％。流式细胞免疫表型：异常细胞占33.08％，表达CD34dim、CD117、CD33、MPO、CD123、CD64，部分表达CD38、CD13、HLA-DR、CD36。RT-PCR技术检测PML-RARα（bcr1）阳性，转录本水平1.88％，PML-RARα（bcr2、bcr3）均为阴性。检测白血病相关FLT3、KIT、NPM1、CEBPA基因突变阴性。WT1阳性率14％。FISH检测PML-RARα阳性，检测位点：15q22/17q21。诊断急性早幼粒细胞白血病（APL）。患者拒绝应用砷剂（ATO）诱导治疗，单用维甲酸（ATRA）20 mg，2次/d口服，20 d后复查血常规：WBC 2.7×10^9^/L，HGB 75 g/L，PLT 152×10^9^/L。凝血系列指标恢复正常。复查骨髓细胞形态学：增生明显活跃，粒红比5.32∶1，早幼粒细胞2％。评价达骨髓形态学缓解，患者拒绝进一步治疗，自动出院后不规则服用ATRA，未规律复查。

患者因“半月前无明显诱因出现头晕、乏力”于2023年1月13日来我院门诊就诊，隐瞒既往诊疗史。血常规：WBC 0.5×10^9^/L，HGB 60 g/L，PLT 17×10^9^/L。门诊以“全血细胞减少”收入院，凝血系列：D-二聚体5 mg/L，余正常。监测凝血系列，纤维蛋白原（FIB）进行性下降，最低1.24 g/L。外周血涂片：白细胞减低；淋巴细胞比例增高，白细胞形态未见明显异常；成熟红细胞形态大小不等；血小板散在少见，形态未见明显改变。骨髓细胞形态学：增生降低（有核细胞分布不均匀），以颗粒异常增多的早幼粒细胞为主，占31.5％。流式细胞免疫表型：原始细胞占有核细胞总数约为25.37％，该群细胞表达CD13、CD33、CD64、CD117；部分表达CD34；少量表达HLA-DR。融合基因分型筛查：RARα融合基因L亚型（bcr1），PML-RARα转录本水平8.963％。染色体核型：46，XX，t(15；17)（q24；q21）/46，XX[1]。二代测序（NGS）检测：WT1突变频率9.5％。RARα基因p.R394G错义突变率11.1％。诊断APL。给予ATO 10 mg/d静脉点滴，联合ATRA 20 mg口服2次/d诱导治疗原发病，低分子肝素钙皮下注射纠正凝血功能异常，间断输注冷沉淀、纤维蛋白原以及血小板治疗，半月余后患者逐渐脱离输注血小板，低纤维蛋白原血症以及D-二聚体增高得到纠正，逐渐停用肝素。但患者白细胞计数始终未见改变，维持在（0.4～0.7）×10^9^/L。2023年2月5日患者出现发热、水肿、体重增加，胸部CT提示胸腔积液，肺感染（图1A、B），超敏C反应蛋白127.8 mg/L，降钙素原0.11 ng/L。微生物动态、病毒抗体系列、血培养及血厌氧菌培养阴性。考虑出现APL分化综合征（DS），暂停ATRA及ATO，给予利尿、抗感染治疗，1周后体温、体重均恢复正常，水肿消散。复查胸部CT提示炎症较前减轻，胸水消退（图1C、D）。患者粒细胞缺乏时间长且合并感染，为避免砷剂的应用加重粒细胞缺乏或感染，2023年2月12日采用单药ATRA治疗，治疗期间白细胞计数仍低，考虑可能与应用ATO后骨髓抑制有关，加用重组人G-CSF 1周。2023年2月24日患者出现高热、呼吸困难、痰中带血、无水肿或体重增加，血常规提示白细胞未见明显上升，复查CT提示肺感染加重，无浆膜腔积液（图2A～C）。超敏C反应蛋白27.1 mg/L，降钙素原0.14 ng/L。微生物动态、病毒抗体系列、血培养及血厌氧菌培养阴性。痰培养：嗜麦芽窄食单胞菌。将ATRA加大至20 mg口服3次/d，停用G-CSF，给予抗感染联合地塞米松5 mg治疗，1周后复查胸部CT提示两肺炎症较前减轻，右肺上叶新发结节影，直径0.8 cm（图2D～G），考虑患者粒细胞缺乏时间长，合并肺真菌感染可能性大，给予伏立康唑经验型抗真菌治疗有效（图2H）。治疗过程中凝血功能逐渐出现异常，D-二聚体进行性增高，再次升至50 g/L，FIB进行性降低，最低降至1.06 g/L，血常规显示血小板计数进行性下降，需间断输注血小板、冷沉淀及纤维蛋白原，继续低分子肝素钙1 ml皮下注射改善凝血，复查骨髓细胞形态学：增生明显活跃，粒红比例28.6∶1，粒系增生以颗粒异常增多的早幼粒细胞为主，占70％。分析初始半个月ATO联合ATRA治疗有效，后单药ATRA治疗近1个月效果差，可能与RARα基因p.R394G错义突变有关，2023年3月1日重新给予单药ATO治疗30 d，患者血常规及凝血系列指标大致恢复正常，复查骨髓细胞形态学达完全缓解（CR）。

**图1:**
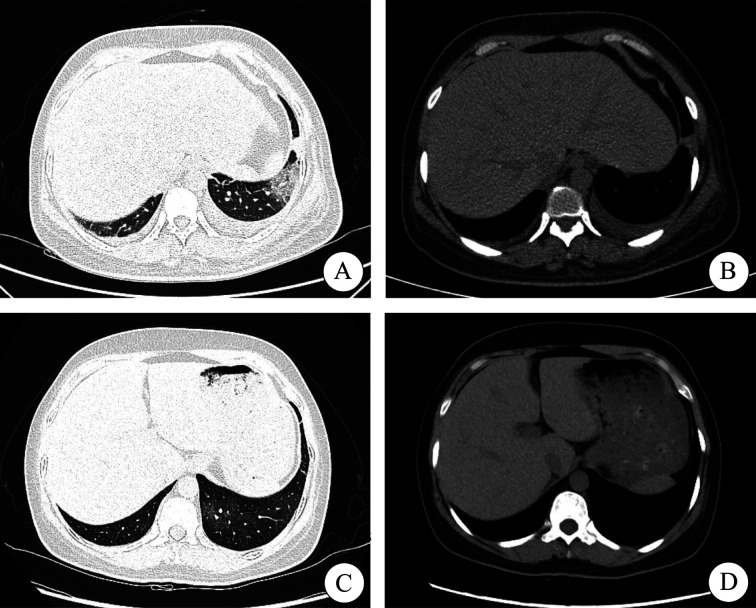
患者急性早幼粒细胞白血病（APL）诱导治疗过程中分化综合征影像学资料 A APL分化综合征发生时胸部CT肺窗； B APL分化综合征发生时胸部CT纵隔窗； C APL分化综合征治疗后胸部CT肺窗； D APL分化综合征治疗后胸部CT纵隔

**图2:**
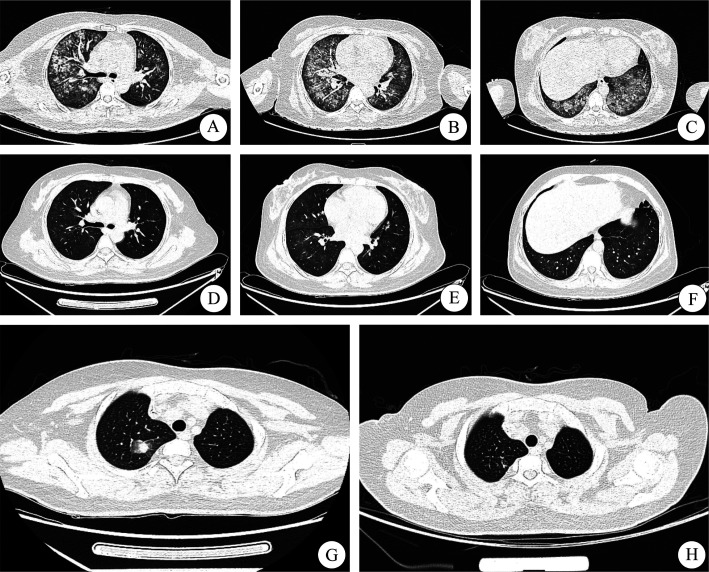
患者急性早幼粒细胞白血病（APL）诱导治疗过程中肺损伤影像学资料 A～C APL诱导治疗中出现肺损伤胸部CT肺窗； D～F APL诱导治疗中抗感染治疗后胸部CT肺窗； G APL诱导治疗中抗感染治疗后新发结节影胸部CT肺窗； H APL诱导治疗中新发结节影抗真菌治疗后胸部CT肺窗

讨论：单药ATRA治疗容易发生维A酸耐药，目前已知的耐ATRA的错义突变有5种：K207N、R272G、G289R、R294W、P407S，均位于RARα配体结合结构域（LBD）中。本例患者隐瞒既往病史，两药诱导治疗半月WBC低下，单药ATRA治疗无效，后期单药ATO治疗1个月后CR，与RARα基因p.R394G错义突变有关，错义突变的产生与患者首次化疗单药ATRA诱导治疗及后续治疗不规律有关。p.R394G是目前新发现的RARα配体结合位点的点位突变，突变同样发生在LBD区。本例患者治疗后期发生重症肺感染，给予抗感染治疗的同时加用糖皮质激素抗炎，可能与诱导治疗过程中应用G-CSF诱发炎性因子风暴有关，在诱导治疗时不建议应用G-CSF。

